# Assessing microplastics contamination and characteristics in organic soil amendments in the Greater Accra Metropolitan Area of Ghana

**DOI:** 10.1016/j.heliyon.2024.e40882

**Published:** 2024-12-04

**Authors:** Benedicta Yayra Fosu-Mensah, Nathanael Nii-Odai Laryea, Daniel Darko, Michael Mensah

**Affiliations:** a Institute for Environment and Sanitation Studies (IESS), College of Basic and Applied Sciences (CBAS), University of Ghana, P. O. Box 209, Legon, Accra, Ghana; b Department of Business Administration, University of Professional Studies, Accra, Ghana

**Keywords:** Municipal solid waste compost, Sewage sludge, Polymers, Plastic waste, Microplastics

## Abstract

The study examines the increasing use of organic soil amendments (OSA) due to declining soil fertility and the high cost of synthetic fertilizers, alongside growing concerns about microplastics (MPs) accumulating in soil, which negatively impact soil, crop, and food quality. This research assessed the presence and characteristics of microplastics in Municipal Solid Waste Composts (MSWC) and dry sewage sludge (DSS) within the Greater Accra Metropolitan Area (GAMA) of Ghana. The study analyzed two sources of MSWC (MSWC 1 and MSWC 2) and two sources of DSS (Sludge 1 and Sludge 2), with five samples each, for microplastic concentrations. A reference soil sample, collected from a depth of 0–25 cm, was also tested. The microplastics were extracted using acid digestion (30 % H_2_O_2_ at 70 °C), density separation with a ZnCl_2_ solution, and vacuum filtration. Results revealed that Sludge 1 had the highest concentration of microplastics (4316 ± 968 MP kg^−1^), followed by MSWC 1 (3572 ± 1196 MP kg^−1^), MSWC 2 (3104 ± 418 MP kg^−1^), and Sludge 2 (2024 ± 562 MP kg^−1^). The soil sample had the lowest concentration of 232 ± 62 MP kg^−1^. Statistical analyses (Kruskal Wallis and Dunn's multiple comparisons) showed significant differences (p < 0.05) in microplastic concentrations among the samples. The composition of microplastic polymers varied among the samples. The soil sample predominantly contained cellophane (91.67 %) and polyvinyl propionate (8.33 %). MSWC 1 contained urethane alkyd (31.11 %), polyethylene (26.67 %), and polyester (20 %), while MSWC 2 had polyethylene (24.10 %), polyester (20.48 %), cellophane (18.07 %), and polypropylene (15.66 %). Sludge 1 was dominated by polyethylene (35.29 %), polypropylene (30.25 %), cellophane (15.13 %), and urethane alkyd (11.76 %), whereas Sludge 2 mainly contained polyester (42.86 %), cellophane (23.21 %), urethane alkyd (21.43 %), and polyethylene (12.50 %). Microplastics were prevalent in the MSWC and sewage sludge which were obtained from the GAMA, with significantly higher concentrations than those in the soil samples. Further research is needed to develop strategies to mitigate microplastic pollution in OSAs to improve soil health.

## Highlights

• Sludge 1 had the highest microplastic (MP) concentration (4316 MP kg⁻^1^).
• Soil had the lowest microplastic concentration (232 MP kg⁻^1^).
• Significant differences were found in microplastic levels across all samples.
• Different sources showed varying MP polymer compositions.
• MSWC 1 and Sludge 1 contained high polyethylene and polypropylene percentages.

## 1 Introduction

Plastics have become integral to everyday life, driven by their cost-effective, durability, lightweight, and adaptable use in diverse products [1,2]. The uses of plastics range from simple household items to advanced uses in agriculture, textile, and aerospace industries [3]. The extensive use of plastics reflects high demand and surge in production since the 1950s [4]. Global plastic production surged from 1.5 million metric tons in 1950 to nearly 391 million metric tons in 2021 and is projected to reach 590 million metric tons by 2050 [5]. The use of plastics results in plastic waste, which contains dangerous pollutants that persist in the environment.

Plastics are non-biodegradable, and they can disintegrate into tiny particles (<5 mm) called microplastics [6,7]. The microplastics impact through various pathways [8]. For instance, modern waste treatment facilities use municipal solid waste (MSW), sewage, or liquid waste as feedstock to produce organic soil amendment products. However, these feedstocks contain microplastics, which often escape filtration during treatment and production of organic soil amendment products such as compost and sewage sludge. When compost and sewage sludge are applied to enrich soil, debris of microplastics is inadvertently introduced, thereby contaminating the soil. Food crops absorb these microplastics (polymers) from the soil, posing food safety and public health risks when consumed [6,7].

Globally, approximately 79 % of plastic waste ends up in landfills, with only 9 % recycled and 12 % incinerated [6,9]. In Ghana, about 60 % of municipal solid waste collected is sent to landfills [10]. Specifically, in the Greater Accra region of Ghana, approximately 14 % (320.79 metric tons) of the daily municipal solid waste (2291.39 metric tons) is composed of plastics [11,12]. Weather-related actions, such as pressure from waste piles and exposure to ultraviolet radiation, stimulate the degradation of the plastic waste to form macroplastics, microplastics, and nanoplastics [13,14]. Furthermore, runoff from landfills, littered areas, and wastewater from washing plastic-based fabrics (polyester clothes) and other items predispose liquid waste to microplastic debris [10,15]. As a result, biosolids such as sludge from sewage liquid and solid wastes facilitate the spread of microplastics [[16], [17], [18]]. Microplastic particles themselves act as carriers of pollutants like heavy metals, dioxins, polychlorinated biphenyls (PCBs), and persistent organic pollutants (POPs) due to additives added during plastic production [18]. These constituent additives are released into the environment as microplastics age, oxidize, and degrade [19], providing suitable conditions and a high surface area for the adsorption (accumulation) of pollutants like heavy metals [20]. In addition to chemical toxicity, microplastics can harm soil organisms when ingested [21]. Continuous application of commercially produced MSWC and sludge-containing plastics as soil amendments may lead to a buildup of microplastics and associated pollutants in agricultural soils [22,23]. This accumulation could affect soil health and compromise the quality and safety of food produced in fields where MSWC and sludge are applied [8,19,24]. Microplastics can alter the physical structure of soils by affecting the aggregation of soil particles. This can lead to changes in soil porosity, water retention, and aeration, which are critical for healthy plant growth [25,26]. Similarly, microplastics can disrupt microbial communities, potentially reducing soil fertility and affecting crop yields [27]. Nawab et al. [28] reported that microplastics can adsorb harmful chemicals, such as pesticides and heavy metals, acting as carriers that introduce these pollutants into the food chain. This can lead to contamination of crops and pose risks to food safety. The ingestion of microplastics by humans, either directly through contaminated food or indirectly through environmental exposure, poses potential health risks. These include physical harm from the particles themselves and chemical toxicity from the pollutants they carry.

Unfortunately, the presence and characteristics of microplastics and related pollutants in terrestrial ecosystems in Africa, particularly Ghana, have not been explored as extensively as in marine ecosystems [20]. In Ghana, organic amendments, such as compost and manure, are widely used in agriculture, and the potential contamination of these materials with microplastics is a growing concern. The presence of microplastics in soils has the potential to undermine sustainable agriculture, pose risks to food safety, and contribute to environmental degradation. Consequently, the occurrence of microplastics in agricultural soils in Ghana and organic fertilizers such as MSWC and sludge as potential sources have not been adequately understood [29]. To address the existing research gap, this study aims to quantify the abundance and characterize the properties of microplastics present in municipal solid waste compost (MSWC) and dry sewage sludge, commonly utilized as organic soil amendments in the Greater Accra Metropolitan Area of Ghana.

## 2 Material and methods

### 2.1 Study area

**Fig. 1:**
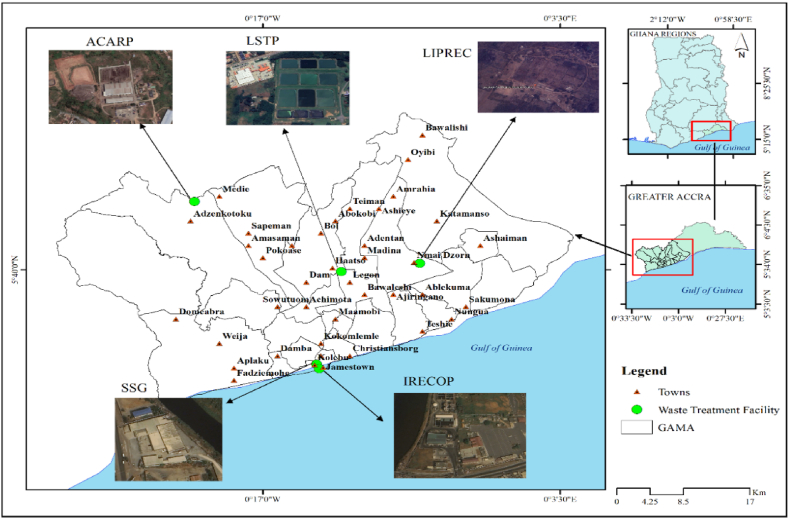
Map of the study area.

The study was conducted on selected organic soil amendment products in the Greater Accra Metropolitan Area (GAMA), (Fig. 1). The GAMA lies between latitude 5.5893° N, and longitude 0.1819° W in the Greater Accra region of Ghana. Accra is the capital of Ghana, and the region's average daily MSW generation is about 0.42 kg per capita, and the region produces about 2291.39 metric tons (Mt) of MSW daily. Most of the MSW comes from GAMA due to its heavily populated commercial centers such as Osu, Kaneshie, Markola, Kantamato, Circle, Dansoman, Madina, Adentan, and their residential areas. The high population density increases waste generation, including plastic waste, which can break down into microplastics. This makes GAMA a critical area for studying microplastic contamination. In addition, the area faces significant challenges in waste management, including inadequate infrastructure and improper disposal methods. Organic waste, often used as organic amendments (e.g., compost or manure), may contain microplastics due to contamination from improperly disposed plastic waste. Given GAMA's significant urban agriculture activities, there is a high likelihood that organic amendments could be contaminated with microplastics, making it an ideal study area to assess this contamination.

About 14 % (320.79 Mt) of the MSW contains plastic materials, [11,12]. Plastic materials include polyethylene bags, food containers, packing materials, bottles, plastic pipes, containers, window frames, flooring materials, and plastic components of textiles, paints, phones, vehicles, and computers, may contain different microplastic polymers.

### 2.2 Research design

The study was designed as comparative experimental research to quantify, characterize and compare polymers of microplastics from different types of organic soil amendment products in GAMA. Two sources of Municipal Solid Waste Composts (MSWC 1 and MSWC 2) and dry sewage sludge (Sludge 1 and Sludge 2) were used as organic soil amendments relative to a reference soil sample. In total, 25 samples were collected in 2022, comprising five samples each of MSWC 1 and MSWC 2, five samples each of dry sludge1 and Sludge 2, and five soil samples.

### 2.3 Sampling of the reference soil and organic soil amendments

The soil samples (Soil) were obtained from fallow land at the University of Ghana's Livestock and Poultry Research Center (LIPREC) in the Adentan Municipality of GAMA. The fallow land was divided into five quadrats. Five soil samples were taken randomly at 0–25 cm depth in each quadrat, using a soil auger, and composited into a glass beaker. Sub-samples of each composited soil sample were taken and well-labeled into a glass bottle**.**

The MSWC 1 originated from Accra Compost and Recycling Plant (ACARP) Ltd. Similarly, MSWC 2 originated from Integrated Recycling and Compost Plant (IRECOP) Ltd. Both plants are major commercial compost producers in Ghana, and their products are available in 50 kg bags on the open market. The composts were produced from mixed organic and non-organic waste collected and sorted later at the production site.

To prepare the compost samples from MSWC 1, samples were taken from five bags, combined in a glass beaker, and thoroughly mixed to create a composite. A sub-sample of this composite was then placed into a glass bottle and labeled. This procedure was repeated with another set of five bags, resulting in five sub-sampled composts, each stored in a labeled glass bottle. The same sampling method was applied to obtain five samples from MSWC 2.

Samples of dry sewage sludge were obtained from two locations: Sewage Systems Ghana (SSG) Ltd. at Korley Gonno (Sludge 1) and the Legon Sewage Treatment Plant (LSTP) near the University of Ghana Botanical Garden (Sludge 2). At both sites, the compost heaps were divided into five sections. From each section, five samples were taken and combined to form a composite sample. A sub-sample of each composite was then placed into a glass bottle and labeled. This gave five composite samples for each type of sludge (Sludge 1 and Sludge 2). All 25 samples were transported to the Ecological Laboratory of the University of Ghana for processing. The processing includes air-drying in a glass bottle at room temperature for a week and then pulverization using a procyclin mortar and pestle [30,31]. The pulverized samples were sieved through a 5 mm stainless steel mesh to remove leaves, roots, and debris [32]. A smaller sub-sample was then taken from each processed sample for microplastic extraction at the Marine and Fisheries Science Department (MFSD) of the University of Ghana. At the laboratory, these processed samples were further divided into five replicates, each for microplastic analysis [33,34].

### 2.4 Extraction of microplastics in MSWC and sludge

The samples were digested using the Wet Digestion Method. One gram (1 g) of each sample was weighed into a digestive tube and digested in a mixture of 10 mL per-chloric acid (HClO_4_ – 78 %), 10 mL sulphuric acid (H_2_SO_4_ – 38 %), and 0.2 ml hydrogen peroxide (H_2_O_2_ – 30 %) [31]. The mixture was then heated in a block digester for 1 h at 200 °C until the solution turned colourless. The digest was allowed to cool and filtered into a 100 mL conical/volumetric flask using Watman number 42 filter paper. The solution was made to 100 mL by topping up with distilled water.

### 2.5 Chemical sample preparation and purification

Two hundred and 50 g (250 g) of soil, compost, and sewage sludge samples were placed in drying trays lined with aluminum foil. To remove organic matter and facilitate effective microplastic extraction and analysis, 50 g (50 g) of the dried samples were purified using the wet digestion approach. Thirty mills (30 mL) of 30 % hydrogen peroxide (H_2_O_2_) and 70 mL of distilled water were added to the 50 g sample in a beaker and heated on a hot plate at 60 °C for 2 h. The solution was subsequently incubated in an oven at 60 °C for 24 h. This process allowed the solution to evaporate, leaving behind plastic particles and other solid matter present in the sample [35].

### 2.6 Extraction of microplastics

Microplastics were isolated from the sample through density separation and filtration [30,36]. A concentrated zinc chloride (ZnCl_2_) salt solution was prepared by dissolving 450 g of the salt in 1L of distilled water for density separation of plastic particles. The ZnCl_2_ solution was added to the digested sample, stirred with a stirring rod for 5 min, and kept overnight to allow particles in suspension to settle while the plastic particles floated. The supernatant was decanted into a clean glass beaker and filtered through a glass microfiber filter ((GF 3 grade (0.6 μm)) with a vacuum filter [20,29]. This type of filter is known for its high retention efficiency for fine particles, making it suitable for filtering small particulates before further analysis. The glass microfiber filters were kept in a Petri dish and placed in an oven at 50 °C for 15–20 min to dry the filters before optical analysis.

### 2.7 Microplastic identification, characterization, and quantification

This analytical process involved optical analysis to identify microplastic characteristics, distribution, and quantities. The process employed microscopy analysis, using a stereomicroscopes [21,37] to quantify the microplastic polymers. All glass microfiber filters in the Petri dishes underwent microscopic analysis at the MFSD Laboratory, using an AS ONE Zoom Stereomicroscope (model: CP745LED Trinocular, Japan) equipped with an LC-15 LCD HDMI Digital Camera from LABOMED INC, USA. The observed microplastics were counted, recorded, and categorized based on shape (fibre, film, fragment, foam, pellet) [30] and colour (red, blue, black, transparent, yellow, green, and purple). Size estimation was conducted using Cloud 1.0 by clicking and dragging the cursor from tip to tip of the observed microplastics.

A 10 % of plastic particles from each glass microfiber filter per sample were placed in a Petri dish and forwarded to the University of Ghana's Department of Chemistry Laboratory for polymer-type identification. Fourier Transform Infrared (FTIR) spectroscopy, conducted with PerkinElmer Spectrum Two, measured the spectra reflectance of the selected plastic particles [38]. The output spectra were then analyzed using OMINIC software (version 9.0) to identify the polymers present in the samples, comparing the peaks with those in a polymer reference library [39]. Based on peak comparisons, the software determined the polymer with a minimum resemblance of 70 %.

### 2.8 Quality control

To control the risk of contamination throughout the process, non-plastic materials were used for the study such as wearing of cotton lab coat. Glassware was used to store and transport the samples to the laboratory. Only glass wares were used throughout the extraction procedures [40,41]. Aluminum foil was used to cover samples throughout the processing and extraction. To monitor potential background contamination during sample treatment, reagent blanks were processed alongside each batch of samples. Procedural blanks (*n* = *3*) were set up by exposing three beakers containing Zn_2_Cl solution close to the working area in the lab and analyzed for air-borne microplastics within the laboratory [42]. In addition, five randomly selected glass microfiber filter papers were selected, observed under a stereo microscope, and burned at 450 °C for 2 h. The filter papers were observed under the microscope again to see if the suspected plastics had disappeared after burning. All experiments in the laboratory were carried out in an ESCO laminar flow cabinet. The limit of detection (LOD) and quantification (LOQ) was calculated as 3 and 10 times the signal-to-noise ratio at the lowest measurable concentration, respectively.

### 2.9 Analysis of data on microplastics

Statistical analyses were performed using SPSS 21.0. Descriptive statistics were used to determine the occurrence (frequency) and distribution (quantity) of various microplastic polymers. Kruskal-Wallis test was done using the GraphPad Prism 8.0.2 to test for significant differences in the mean number of polymers obtained from the samples, based on a 95 % confidence interval (p < 0.05). Dunn's multiple comparisons test was performed to compare the mean abundance between samples [43,44].

## 3 Results

### 3.1 Physicochemical properties of samples

Table 1 presents the physicochemical properties of soils, Municipal Solid Waste Compost (MSWC), and Sewage Sludge. From the results, MSWC 2 recorded the highest pH level (8.3 ± 0.045), while the lowest pH was observed in sludge 2 (5.0 ± 0.114). In terms of electrical conductivity (EC), sludge 2 recorded the highest value (1.899 ± 0.001 dS/m), whereas the soil had the lowest EC (0.288 ± 0.079 dS/m). The organic carbon content was highest in sludge 1 (29.360 ± 0.247 %) and lowest in the soil (1.530 ± 0.053 %). The soil also had the lowest total nitrogen content (0.070 ± 0.002 %), while sludge 1 recorded the highest (31.815 ± 0.531 %). Total phosphorus levels ranged from 387.734 ± 24.453 mg/kg in the soil to 4868.982 ± 54.879 mg/kg in MSWC 2. Various concentrations of Cr, Cd, Pb, and Ni were detected across all samples.

**Table 1:** Physicochemical properties of soils, Municipal Solid Waste Compost and Sewage Sludge.

**Physicochemical Properties**	**Soil**	**----- Municipal Solid Waste Compost -----**		**--------- Sewage Sludge ---------**	
		**MSWC 1**	**MSWC 2**	**Sludge 1**	**Sludge 2**
Bulk density	1.56 ± 0.02				

Particle size by weight (%)					

Sand	21.3				
Silt	42.7				
Clay	36.0				

Exchangeable bases [cmol(+)/kg]					

Calcium (Ca)	5.997 ± 0.960				
Magnesium (Mg)	4.143 ± 1.985				
CEC	11.491 ± 3.081				

pH	7.1 ± 0.259**∗**	7.9 ± 0.084**∗∗**	8.3 ± 0.045**∗∗**	6.3 ± 0.089**∗∗**	5.0 ± 0.114**∗∗**
Electrical Conductivity (dS/m)	0.288 ± 0.079	0.868 ± 0.045	1.328 ± 0.005	1.899 ± 0.001	0.670 ± 0.002
Organic Carbon (%)	1.530 ± 0.053	5.408 ± 0.121	6.890 ± 0.247	29.360 ± 0.247	17.916 ± 0.246

Total Nitrogen (%)	0.070 ± 0.002	5.201 ± 0.318	3.066 ± 0.212	31.815 ± 0.531	9.653 ± 0.106
Total Phosphorus (mg/kg)	387.734 ± 24.453	4786.436 ± 42.263	4868.982 ± 54.879	24614.441 ± 49.892	24805.004 ± 29.602

Heavy Metals (mg/kg)					

Chromium (mg/kg)	22.532 ± 1.080	24.660 ± 5.824	19.870 ± 3.543	53.330 ± 2.661	22.380 ± 3.889
Cadmium (mg/kg)	2.670 ± 0.262	1.620 ± 0.358	1.520 ± 0.798	3.010 ± 0.724	1.340 ± 0.417
Lead (mg/kg)	10.614 ± 1.561	55.566 ± 7.248	40.730 ± 6.384	47.120 ± 6.043	28.530 ± 10.451
Nickle (mg/kg)	21.104 ± 3.033	22.442 ± 0.050	7.100 ± 0.167	6.132 ± 0.137	8.498 ± 0.186

### 3.2 Abundance of microplastics in organic soil amendments

The results showed that microplastics were present in approximately 98 % of all the analyzed samples (Table 2). A total of 662.4 microplastic particles were identified in the samples. Soil, MSWC 1 (ACARP), Sludge 2 (LSTP), MSWC 2 (IRECOP), and Sludge 1 (SSG) accounted for approximately 1.75 %, 26.96 %, 15.28 %, 23.43 %, and 32.58 % of the total microplastic abundance respectively. Sludge 1 recorded the highest number of microplastics with a mean value of 215.8 ± 48.40 microplastic particles (MPs) per 50 g of sample, followed by MSWC 1, with values of 178.6 ± 59.79 MPs per 50 g of sample. Microplastic particles in MSWC 2 amounted to 155.2 ± 20.89 MPs per 50 g of sample, with Sludge 2 amounted to 101.2 ± 28.17 MPs per 50 g of sample while microplastic concentration in the soil (LIPREC) was 11.6 ± 3.05 MPs per 50 g of sample. These amounts are equivalent to 4316 MP kg^−1^ in Sludge 1, 2024 MP kg^−1^ in Sludge 2, 3572 MP kg^−1^ in MSWC 1, and 3104 MP kg^−1^ in MSWC 2.

**Table 2:** Abundance of microplastics in organic soil amendments.

**Types of organic soil amendment products**	**Number of samples**	**Quantities of microplastics (MPs)**	
		**Mean (MPs per 50 g)**	**MPs kg**^**−**^**^1^**
MSWC 1 (ACARP)	5	178.6 ± 59.8	3572 ± 1196
MSWC 2 (IRPCOP)	5	155.2 ± 20.9	3104 ± 418
Sludge 1 (SSG)	5	215.8 ± 48.4	4316 ± 968
Sludge 2 LSTP	5	101.2 ± 28.2	2024 ± 562
Soil (LIPREC)	5	11.6 ± 3.1	232 ± 62

Total (combined)	25	662.4	13248

MSWC 1 is organic compost from Accra Compost and Recycling Plant Ltd. (ACARP); MSWC 2 is organic compost from Integrated Recycling and Compost Plant Ltd. (IRECOP); Sludge 1 is dry sewage sludge from Sewage Systems Ghana Limited (SSG); Sludge 2 is dry sewage sludge from Legon Sewage Treatment Plant (LSTP): Soil is composite soils from Livestock and Poultry Research Centre (LIPREC) of the University of Ghana.

**Fig. 2:**
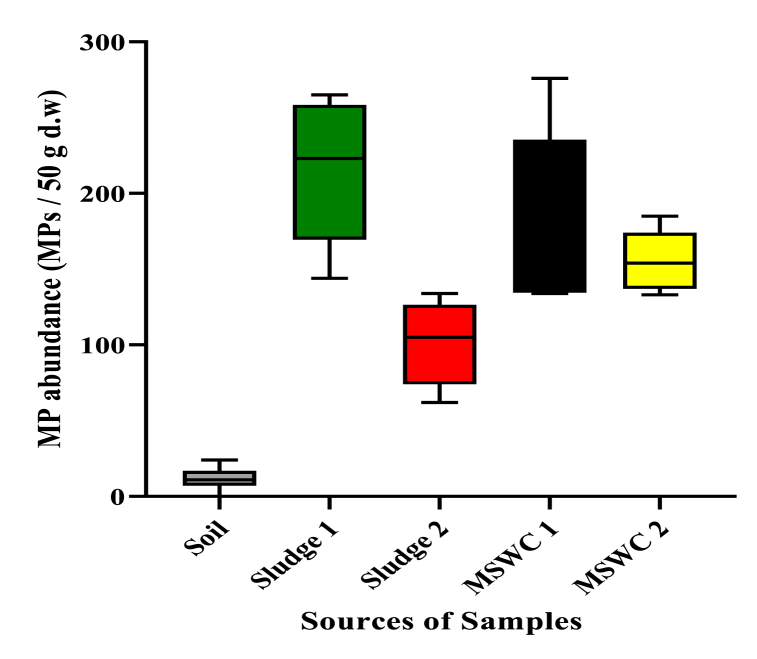
Distribution of microplastics at individual sampled sources.

The microplastic abundance (Fig. 2) ranged from 0 to 24 MPs per 50 g for soil samples, 144–265 MPs per 50 g for sludge 1, 62–134 MPs 50 g for sludge 2, 134–276 MPs per 50 g for MSWC 1 and 133–185 MPs per 50 g for MSWC 2.

### 3.3 Characteristics of microplastics in soils and soil amendments

#### 3.3.1 Morphological distribution of microplastics in samples

**Fig. 3:**
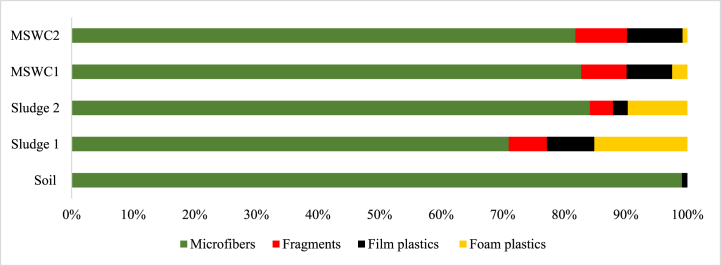
Percentage distribution of the microplastic shapes across the sampled sources.

Four microplastic morphology (shape) types were observed in all the samples (Fig. 3), which were microfibers, fragments, film plastics, and foam. In the soil samples, only microfibers, film plastics, and foam plastics constituted 99.13 % and 0.87 % of the recorded microplastic abundance. The constitution of microfibers, fragments, film plastics, and foam plastics in the microplastics abundance recorded for sludge 1 was 70.99 %, 6.20 %, 7.69 %, and 15.10 %, respectively. Similarly, in the sludge 2 samples, microfibers, fragments, film plastics, and foam plastics constituted 84.19 %, 3.75 %, 2.37 %, and 9.68 %, respectively, of the microplastics abundance recorded. For the MSWC 1 samples, microfibers, fragments, film plastics, and foam plastics constituted 82.75 %, 7.39 %, 7.39 %, and 2.46 % of microplastic abundance recorded, respectively. Finally, the composition of microfibers, fragments, film plastics, and foam plastics in MSWC 2 accounted for 81.83 %, 8.38 %, 9.02 %, and 0.77 % of the microplastics abundance recorded, respectively (Fig. 3).

Results of the Kruskal-Wallis's test showed a statistical difference (*p* < 0.05) in the abundance of microplastics across sampling sources. Significant differences in microplastic abundance were observed within sample sources in only three comparisons. Results from Dunn's Multiple Comparison Test (Table 3) showed significant differences (*p* < 0.05) in microplastic occurrence between (i) Sludge 1 and Soil, (ii) MSWC 1 and Soil, and (iii) MSWC 2 and Soil.

**Table 3:** Dunn's multiple comparison tests between sample sources.

**Dunn's multiple comparisons test**	**Mean rank diff.**	**F-probability**	**Adjusted P Value**
Sludge 1 vs. Sludge 2	12.6	NS	0.8811
Sludge 1 vs. MSWC 1	3.7	NS	>0.9999
Sludge 1 vs. MSWC 2	5.3	NS	>0.9999
Sludge 1 vs. SOIL	25.4	∗∗∗	0.0001
Sludge 2 vs. MSWC 1	−8.9	NS	>0.9999
Sludge 2 vs. MSWC 2	−7.3	NS	>0.9999
Sludge 2 vs. SOIL	12.8	NS	0.2842
MSWC 1 vs. MSWC 2	1.6	NS	>0.9999
MSWC 1 vs. SOIL	21.7	∗∗∗	0.002
MSWC 2 vs. SOIL	20.1	∗∗∗	0.0058

∗∗∗ is significantly different, NS is not significant. The difference was assumed significant when the adjusted P value generated is less than the α-value = 0.05. MSWC 1 is organic compost from Accra Compost and Recycling Plant Ltd. (ACARP); MSWC 2 is organic compost from Integrated Recycling and Compost Plant Ltd. (IRECOP); Sludge 1 is dry sewage sludge from Sewage Systems Ghana Limited (SSG); Sludge 2 is dry sewage sludge from Legon Sewage Treatment Plant (LSTP): Soil is composite soils from Livestock and Poultry Research Centre (LIPREC) of the University of Ghana.

#### 3.3.2 Size range distribution of microplastics in samples

**Fig. 4:**
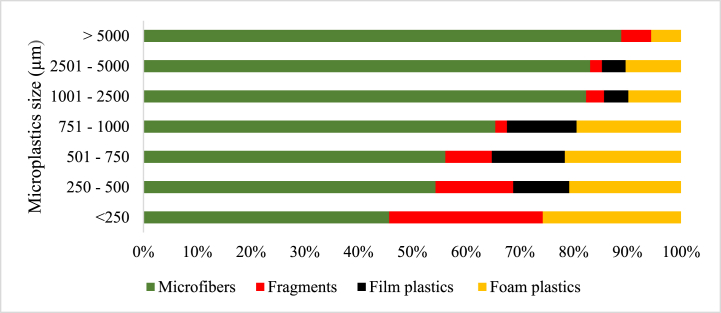
The distribution and size (μm) of microplastics by morphology in Sludge 1.

The size (length) estimation of the microplastics was categorized as < 250 μm, 251–500 μm, 501–750 μm, 751–1000 μm, 1001–2500 μm, 2501–5000 μm and >5 mm (Fig. 4). In the Sludge 1 samples, microfibers recorded the highest abundance in all size categories, followed by foam plastics, which also recorded high numbers in all size categories. The size range >5 mm recorded equal microfibers and fragments. In the <250 μm sizes, fragments recorded a higher percentage abundance. Film plastics were recorded in only five size ranges: 250–500 μm, 501–750 μm, 751–1000 μm, 1001–2500 μm and 2501–5000 μm in Sludge 1 samples. The number of foam plastics surpassed that of fragments in all the size ranges they occurred except the 250–500 μm size range, where its abundance was exceeded by that of fragments (Fig. 4).

**Fig. 5:**
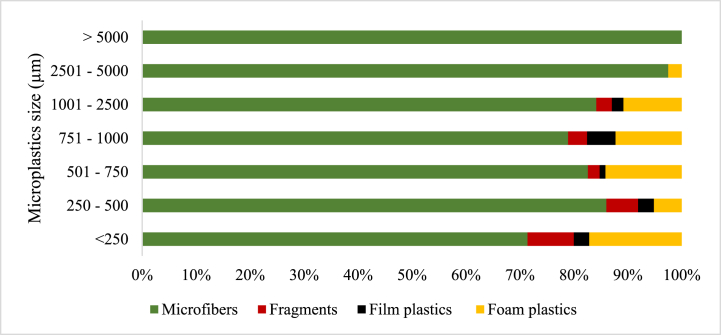
The distribution and size (μm) of microplastics by morphology in Sludge 2.

In sludge 2 samples, microfibers recorded the highest numbers in all size ranges, while foam plastics recorded the second highest numbers at size ranges <250 μm, 501–750 μm, 751–1000 μm, and 1001–2500 μm. Occurrence of fragments and film plastics was observed in only five size ranges: <250 μm, 250–500 μm, 501–750 μm, 751–1000 μm, and 1001–2500 μm with a percentage abundance in fragments exceeding film plastics in all these size ranges except for the 751–1000 μm range (Fig. 5). Microfibers were, however, found to be the most dominant in all individual sizes and ranges.

**Fig. 6:**
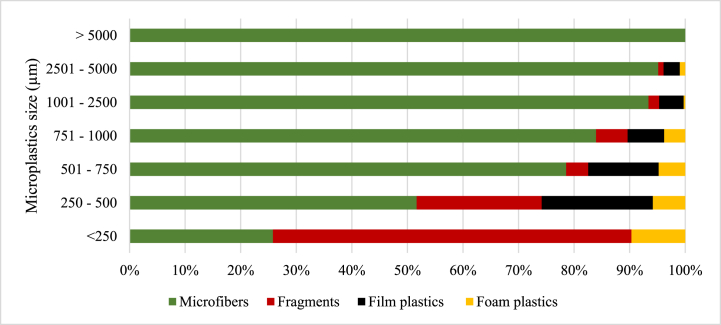
The distribution and size (μm) of microplastics by morphology in MSWC1.

In MSWC 1 samples, microfibers recorded the highest numbers in all size ranges except for the <250 μm size category, where fragments had the highest percentage abundance. The fragments and foam plastics occurred in all sizes, but the size range was >5 mm, and film plastics occurred in 5 out of the seven (7) size ranges except for the >5 mm and <250 μm size ranges. However, foam plastics in the MSWC 1 samples recorded the least percentage abundance in all size ranges except in the 2501–5000 μm range, which was at par with fragments (Fig. 6).

**Fig. 7:**
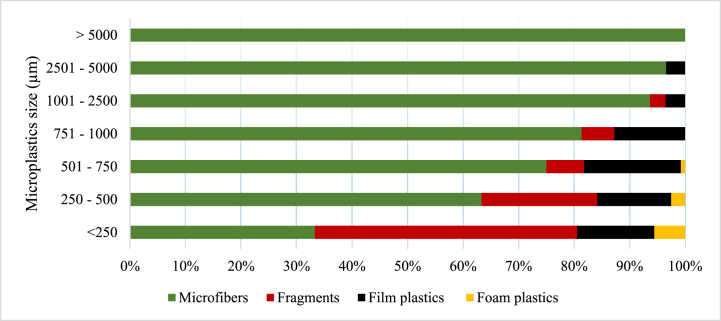
The distribution and size (μm) of microplastics by morphology in MSWC 2.

The percentage abundance of microfibers in MSWC 2 was also found to be the most dominant at the various size ranges except at < 250 μm, where fragments were most predominant. Fragments occurred in five (5) out of the seven size ranges except for the 2501–5000 μm and the >5 mm size ranges. Film plastics, however, occurred in all the size ranges except the >5 mm size range. Besides microfibers, film plastics were the second dominant category in abundance in all the size ranges of the MSWC 2 samples except in the <250 μm and 251–500 μm categories, where fragments recorded the third highest abundance after microfiber and fragment, respectively. Foam plastics occurred in only three (3) sizes: <250 μm, 251–500 μm and 501–750 μm. However, the percentage of foam plastics was the lowest in all the size ranges for MSWC 2 (Fig. 7).

**Fig. 8:**
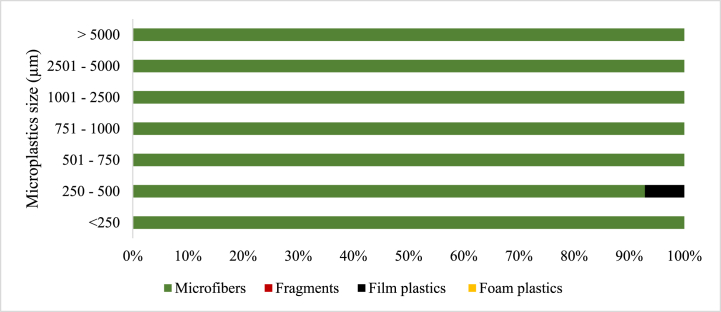
The distribution and size (μm) of microplastics by morphology in Soil.

In soil samples, microfibers were the most dominant in almost all size ranges except for the 250–500 μm size range, where the number of film plastics exceeded that of microfiber (Fig. 8). There was no occurrence of fragments and foam plastics in any of the size ranges of microplastics observed in the soil samples.

#### 3.3.3 Microplastic polymer distribution in soil and soil amendments

Supplementary Figs. 9–12 in appendix shows some polymer peaks obtained from OMNIC polymer analysis of microplastics. Polymer peaks obtained from OMNIC polymer library after further analysis of the Fourier Transformed Infrared Spectrometry (FTIR) output for MSWC 2 showed that eight polymer types were present. These included urethane alkyd, polystyrene, polypropylene, polyethylene, cellophane, polyester, polyvinyl acetate, and polyvinyl propionate. Amongst these, the dominant polymers in the MSWC 2 sample were polyethylene (24.10 %) and polyester (20.48 %). Cellophane and polypropylene followed closely, contributing 18.07 % and 15.66 %, respectively. Urethane alkyd and polystyrene were next, contributing 7.23 % each to microplastic abundance, while polyvinyl acetate and polyvinyl propionate made the lowest percentage composition to microplastics with 3.61 % each. However, MSWC 1 yielded seven polymer types made of urethane alkyd, polypropylene, cellophane, polystyrene (Supplementary Fig. 9), polyethylene, polyvinyl acetate, and polyester. Urethane alkyd was the most dominant polymer in MSWC 1, contributing 31.11 %. This was followed by polyethylene and polyester, which account for 26.67 % and 20.00 % of microplastic composition, respectively. The least contribution to microplastic abundance in the MSWC 1 sample was made in descending order of polypropylene, polystyrene, and polyvinyl acetate 6.67 %, 4.44 %, and 3.33 %, respectively (Table 4).

**Table 4:** Distribution of microplastic polymers in samples.

**Microplastic Polymers**	**Quantity**	**%**	**Quantity**	**%**
	**MSWC 2**		**MSWC 1**	
Urethane Alkyd	6	7.23	28	31.11
Polypropylene	13	15.66	6	6.67
Cellophane	15	18.07	7	7.78
Polystyrene	6	7.23	4	4.44
Polyethylene	20	24.10	24	26.67
Polyvinyl Acetate	3	3.61	3	3.33
Polyester	17	20.48	18	20.00
Polyvinyl Propionate	3	3.61	–	–
**Total**	**83**		**90**	

	**Sludge 1**		**Sludge 2**	

Urethane Alkyd	14	11.76	12	21.43
Cellophane	18	15.13	13	23.21
Polyethylene	42	35.29	7	12.50
Polyester	6	5.04	24	42.86
Polyvinyl Propionate	3	2.52	–	–
Polypropylene	36	30.25	–	–
**Total**	**119**		**56**	

	**Soil**			

Cellophane	11	91.67		
Polyvinyl Propionate	1	8.33		
**Total**	**12**			

MSWC 1 is organic compost from Accra Compost and Recycling Plant Ltd. (ACARP); MSWC 2 is organic compost from Integrated Recycling and Compost Plant Ltd. (IRECOP); Sludge 1 is dry sewage sludge from Sewage Systems Ghana Limited (SSG); Sludge 2 is dry sewage sludge from Legon Sewage Treatment Plant (LSTP): Soil is composite soils from Livestock and Poultry Research Centre (LIPREC) of the University of Ghana.

Similarly, six polymer types were identified in Sludge 1, covering urethane alkyd, cellophane, polyethylene (Supplementary Fig. 10), polyester, polyvinyl propionate, and polypropylene (Supplementary Fig. 11).

The polymer contribution to microplastic abundance in the Sludge 1 sample was dominated by polyethylene (35.29 %) and polypropylene (30.25 %). Cellophane and urethane alkyd accounted for the third and fourth highest contributions, with 15.13 % and 11.76 %, respectively, and polyester accounted for 26.67 %. Polyester and polyvinyl propionate contributed the least to microplastic abundance with 5.04 % and 2.25 % compositions, respectively. Similarly, four polymer types were identified in Sludge 2, these were: urethane alkyd, cellophane, polyethylene, and polyester. Polyester was the most dominant, accounting for 42.86 % of microplastics, followed by urethane alkyd and cellophane, contributing 21.43 % and 23.21 % of microplastics, respectively. Polyethylene polymer had the least contribution to microplastics in the Sludge 2 sample, with 12.50 % of microplastics.

Cellophane (Supplementary Fig. 12) and polyvinyl propionate were the only polymers identified in the soil sample. Cellophane dominated microplastic abundance in the soil sample with an overwhelming contribution of 91.67 %, while polyvinyl propionate accounted for only 8.33 % of microplastics (Table 4).

## 4 Discussions

### 4.1 Occurrence of microplastics

The study found microplastics (MPs) in all the Municipal Solid Waste Composts (MSWC 1 and MSWC 2) and dry sewage sludges (sludges 1 and sludges 2) with higher mean concentration (abundance) than the reference soil samples (Soil). The abundance of soil MPs (11.6 ± 3.05 MPs 50 g^−1^; ranged from 0 to 24 MPs 50 g^−1^ at 25 cm soil depth) was higher than previous reports. Liu et al. [45] reported a mean concentration of 84.75 ± 13.22 kg^−1^ and 65.75 ± 13.92 kg^−1^ in surface and deep soils of suburban farmland in Shanghai, respectively. The range of abundance (0–24 MPs 50 g^−1^) of soil microplastics in the current study was also lower than 0–593 MP kg^−1^ in soil samples from floodplains in Switzerland [46]. In Mellipilla, Chile, a higher abundance range (600–10400 MP kg^−1^) was observed in agricultural soil sampled at 25 cm depth [36]. The difference in the mean microplastic abundance may be attributed to land use, soil texture, and management practices. The current soil sample site had animal grazing activities and silty clay loam texture, classified as vertisol, and thus, very compact, with a hard surface and deep cracks when dry [47]. These characteristics, coupled with compaction by livestock, decrease the soil permeability, making it much more difficult for tiny particles like microplastics to penetrate [48]. According to Scheurer and Bigalke [46] hard surface soil facilitates particle transportation, as microplastics on agricultural topsoil may be washed away during heavy rains. Additionally, the low concentration of soil microplastics could be due to low or no application of external inputs like fertilizer and compost, among others, at the current soil sample site. It has been established that repetitive sludge and compost application contribute to high microplastic concentration in soils [26,29,49]. Microplastics (MPs) in compost can degrade its quality and pose ecological risks. MPs can alter compost's structure, reduce nutrient availability, and hinder microbial activity, compromising its effectiveness as a soil amendment [50]. They can also persist in the environment, transferring from compost to soil and potentially entering the food chain through crops. Over time, MPs fragment into smaller particles, increasing their ecological impact by being ingested by soil organisms, disrupting soil biodiversity and function. Additionally, MPs can adsorb and release pollutants like pesticides and heavy metals, exacerbating environmental contamination.

The abundance of microplastics in dry sewage Sludge 2 from Legon Sewage Treatment Plant (LSTP) (2024 ± 562 MP kg^−1^) was lower than Sludge 1 from Sewage System Ghana (SSG) (4316 ± 968 MP kg^−1^). Earlier, Li et al. [51] recorded 29.0 × 10^3^ MP kg^−1^ in sludge samples from Jiangsu Province, 30.7 × 10^3^ MP kg^−1^ in Shandong Province, and 7.7 × 10^3^ MP kg^−1^ at Yunnan Province in China. The vast difference in sludge microplastic concentration in the sites of the present study and the previous ones can be attributed to higher population density and investment in built (fixed) assets as well as less afforested land area in the Jiangsu and Shandong Provinces.

The current study also suggests that the average microplastic concentration of sludge positively correlates with population density and total investments in fixed assets (p < 0.05) while negatively associated with afforested land (p < 0.01). The current sites had lesser population densities than those in the study by Li et al. [51].

Furthermore, the abundance of microplastics in the MSWC 1 from Accra Compost and Recycling Plant (ACARP) Ltd (3572 ± 1196 MP kg^−1^) and MSWC 2 from Integrated Recycling and Compost Plant (IRECOP) Ltd. (3104 ± 418 MP kg^−1^) were relatively high. Gui et al. (2021) recorded 2533 ± 457 MP kg^−1^ and 2267 ± 115 MP kg^−1^ from 2 sample stations. According to Gui et al. [52], microplastic abundance increases during various stages of composting. The microplastic concentration may increase from 800 ± 200 kg^−1^ to 1133 ± 115 kg^−1^ after manual sorting and crushing. When the temperature increased during aerobic decomposition, the concentration rose to 1667 ± 306 MP kg^−1^ [52].

Subsequently, the concentrations rose to 1733 ± 231 MP kg^−1^ and 1933 ± 416 MP kg^−1^ during high-temperature and cooling stages of composing, respectively. Zhang et al. [53] also recorded 14,720 ± 2468 MP kg^−1^, 8600 ± 1428 MP kg^−1^, and 11,640 ± 3565 MP kg^−1^ for composts made from chicken manure, sludge, and domestic waste, respectively. Therefore, the difference in the microplastic abundance between compost samples in the present study and previous studies can be attributed to temperature differential during composting and feedstock quality. Temperature significantly influences the abundance of microplastics (MPs) during composting by affecting both the degradation of organic matter and the fragmentation of plastics. As temperature increases during aerobic decomposition, microbial activity accelerates as well as the breakdown of larger plastic debris into smaller microplastic particles [54].

### 4.2 Characteristics and distribution of microplastics

The present study observed microfibres, fragments, film plastics, and foam plastics across all samples except in soil, where only microfibers and film plastics were recorded. The shapes of microplastics showed that microfibre is the most abundant microplastic form, ranging from 71.0 % to 84.2 % of occurrence, which is in line with Liu et al [45]. and Li et al [51]. who observed 53.3 % macrofibre content in soil samples, with fragment, film, and pellet content being 37.58 %, 6.67 %, and 2.12 %, respectively, which can be attributed to differences in regional microplastic sources.

The current study revealed different sizes of microplastics in all five sample locations ranging from 1001 to 2500 μm, except for Sludge 2 (LSTP), which had “250–500 μm”. In the sludge samples from Sludge 1 (SSG) and Sludge 2 (LSTP), respectively, 928 of 1082 (85.77 %) and 459 of 506 (90.71 %) microplastic particles recorded were ≤2500 μm in size. Sludge 1 (SSG) had 419 (45.15 %) particles of microplastics being 1001–2500 μm in size, while Sludge 2 (LSTP) had 139 (27.47 %) and 136 (26.87 %) particles of microplastics being 1001–2500 μm and 250–500 μm in size, respectively. The statistical difference observed between Sludge 1 and Sludge 2 may be attributed to the origin of plastic waste relative to the location and design of the liquid waste treatment facilities. SSG is located near the highly polluted Korley Lagoon and busy commercial centers of Accra, including populous communities in Accra Central and slums along the coastline at Korley Gonno, Whereas LSTP operates close to the Legon Botanical Gardens, on the University of Ghana campus. The plant serves other well-planned and organized parts of Accra such as Achimota, Presbyterian Boys Senior High School, Ghana Institute of Management and Public Administration, University of Professional Studies Accra, and other suburbs around Legon. These areas have lower population densities, better sanitary conditions, and less pollution than other parts of Accra. The MPs enter sewage sludge primarily through household and industrial effluents, including wastewater from laundry, personal care products, and industrial discharges. Microplastics in dry sewage sludge are generally smaller. The wastewater treatment process, including filtration, captures smaller MPs, resulting in a higher concentration.

Additionally, only liquid waste is channeled through sewage lines to the LSTP compared to SSG, whose plant was designed to allow for dislodged liquid waste from cesspit trucks. Also, trucks transport liquid waste to the SSG from various locations within Accra, where populations are higher with relatively poor sanitary conditions compared to better sanitary conditions of Legon and Achimota environs that is served by the Legon Sewage Treatment Plant [[55], [56], [57]].

### 4.3 Polymer composition of microplastics in samples

The presence of various polymer types in the MSWC, dry sewage sludge, and soil samples had varying proportions. The top three polymers by percentage composition present in MSWC 2 (IRECOP) were polyethylene (24.10 %), polyester (20.48 %), and cellophane (18.07 %), whereas, in MSWC 1 (ACARP), they were urethane alkyd (31.11 %), polyethylene (26.67 %) and polyester (20.00 %). The occurrences of polyethylene and polyester were similar in both MSWC sources, with a slightly higher polyethylene composition in the MSWC 1. Polyethylene is widely used in packaging materials, indicating substantial plastic waste contribution. Similarly, the high percentage of polyester suggests significant textile waste in the compost as polyester is common in textiles. Polyester has been found in significant concentrations in irrigation water, primarily due to the high levels of plastic fibers released from laundry processes [58]. The apparent differences in polymer composition in both samples were higher cellophane occurrence in MSWC 2 and higher urethane alkyd in MSWC 1. The high percentage of Urethane Alkyd (31.11 %) in MSWC 1 suggests that industrial or construction waste, particularly from painted surfaces or coatings, is a significant contributor to this compost. Apart from the cellophane and urethane alkyd variation, the finding of this study agrees with reports of Vithanage et al. [59] which showed that polyethylene and polyester were among the polymer types most dominant in composts from municipal waste sources.

The feedstock for both composts originating from domestic wastes whose sources are different (from varying locations and activities) could explain the variations in the polymer composition of microplastics since it is difficult to attribute the plastic polymer to a particular source [60,61]. At the same time, the similarity in polyethylene and polyester composition indicates a resemblance in activity causing the pollution and polymer sources.

The study revealed that polyethylene (35.29 %), polypropylene (30.25 %), and cellophane (15.13 %) were the most dominant polymers in Sludge 1 (SSG), whereas in Sludge 2 (LSTP) had polyester (42.86 %), cellophane (23.21 %) and urethane alkyd (21.43 %). The occurrence of polyethylene and polypropylene in Sludge 1 and polyester in Sludge 2 in relatively larger proportions is consistent with the evidence of these polymers in sewage sludge from other locations worldwide [62]. They also indicate the origin of the liquid waste [63]. Hatinoglu [62]. opined that the occurrence of these polymers reflects the use patterns of plastics in everyday products, with polyethylene, polypropylene, and polyester emanating from packaging for personal care products, food, and synthetic clothing. Observation of higher proportions of urethan alkyd and cellophane in Sludge 2 (LSTP) gives indications of polymers from oil-based paints/coatings and food wrapping/packaging materials, respectively [60,64,65]. The occurrence of these coatings/paints in Sludge 2 could emanate from the peels/scrapings of old paints from the yearly refurbishment of educational institutions facilities whose liquids waste are channeled to the Legon Liquid Waste Treatment Facility (Sludge 2) through the sewage network system [55,56,66].

Polymer types identified in the soil sample were cellophane (91.6 %) and polyvinyl propionate (8.33 %), which were inconsistent with findings from other agricultural soils. In other studies, polymer types found in agriculture/farm soils include polypropylene, polyethylene, polystyrene, polypropylene, nylon, polyvinyl chloride, and polyether sulfones [67]. For instance, Liu [45]. identified polypropylene (51 %), polyethylene (43 %), and polyether sulfones (6 %) in farm soil in Shanghai, China. In addition, Vollertsen & Hansen [68] reported polyethylene (89 %), nylon (12 %), and polypropylene (1 %) as polymer compositions of background microplastics abundance identified in agricultural soils in Northern Jutland, Denmark.

The high percentage of polyethylene, polyester, and cellophane in MSWC and sewage sludge indicates extensive soil contamination when used as organic amendments in agriculture. Microplastics can alter soil structure, reduce porosity, and affect water retention and nutrient availability, ultimately impacting plant growth, microbial activities, and soil health [[69], [70], [71]]. The unique presence of cellophane and polyvinyl propionate in soil samples indicates contamination from food packaging and construction materials, potentially leading to long-term environmental degradation.

The use of contaminated compost and sludge in agriculture increases the likelihood of microplastics entering the food chain, either through direct ingestion by crops or through soil organisms. Humans can be exposed to these microplastics through the consumption of contaminated food, posing potential health risks, including inflammation in the ovaries, toxicity, and the disruption of biological processes and reproductive systems [72]. Polyethylene and polypropylene, which are commonly used in food packaging, pose ingestion risks. The small size of microplastics allows them to cross biological barriers, potentially accumulating in human tissues and organs, leading to long-term health effects. Furthermore, a recent report by Ref. [73] indicated a reduction in testosterone bioavailability and sperm quality as a result of exposure to microplastics.

## 5 Conclusion

This study confirms the widespread presence of microplastics in Municipal Solid Waste Composts (MSWC) and dry sewage sludge within the Greater Accra Metropolitan Area, with concentrations significantly higher than those found in the reference soil samples. The results reveal that sewage sludge exhibited the highest levels of microplastics, while compost and soil samples show comparatively lower concentrations.

The variations in microplastic abundance across different sludge and compost samples were linked to population density, waste management practices, and composting processes. Furthermore, the size range distribution analysis revealed that microplastics in the studied samples predominantly fell within the smaller size categories (<250 μm to 1001–2500 μm), which have implications for their potential uptake and impacts on biota. The polymer composition analysis revealed that polyethylene and polyester were the most common polymers across various sample types.

The results highlight the need for strategies to mitigate microplastic pollution in organic soil amendments to protect soil health and crop quality.

## CRediT authorship contribution statement

**Benedicta Yayra Fosu-Mensah:** Writing – review & editing, Writing – original draft, Supervision, Methodology, Conceptualization. **Nathanael Nii-Odai Laryea:** Methodology, Formal analysis, Data curation, Conceptualization. **Daniel Darko:** Supervision, Methodology, Conceptualization. **Michael Mensah:** Writing – review & editing, Methodology.

## Data availability

Data will be made available upon request.

## Funding sources

This research work was self-financed.

## Declaration of competing interest

The authors declare that they have no known competing financial interests or personal relationships that could have appeared to influence the work reported in this paper.
