# Supplementary material for: Assessing microplastics contamination and characteristics in organic soil amendments in the Greater Accra Metropolitan Area of Ghana
Source: Heliyon. 2024 Dec 4;10(23):e40882. doi: 10.1016/j.heliyon.2024.e40882 (PMC11666938; doi:10.1016/j.heliyon.2024.e40882)
Supplement: Multimedia component 1 [file mmc1.docx]

Supplementary Figures

**
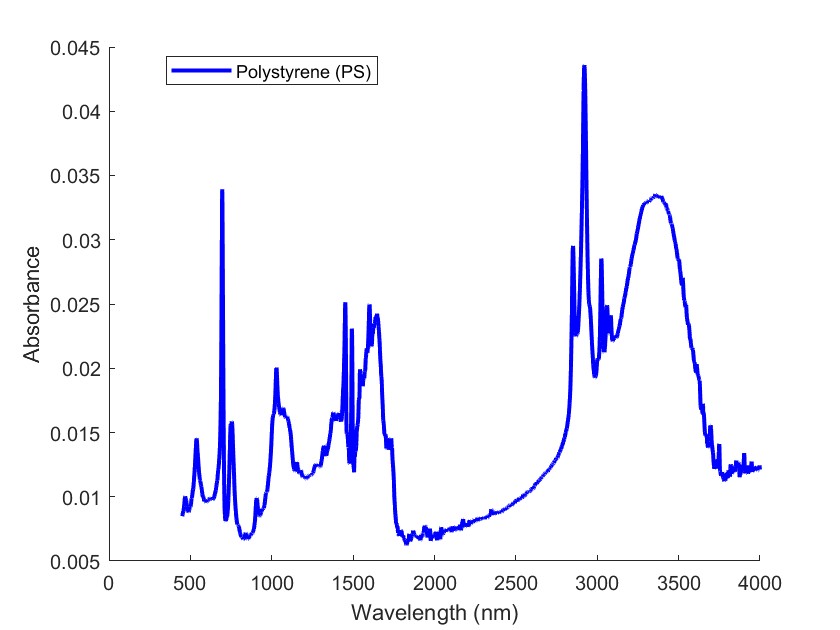
**

Supplementary Figure 9 Polystyrene peaks of MSWC 1


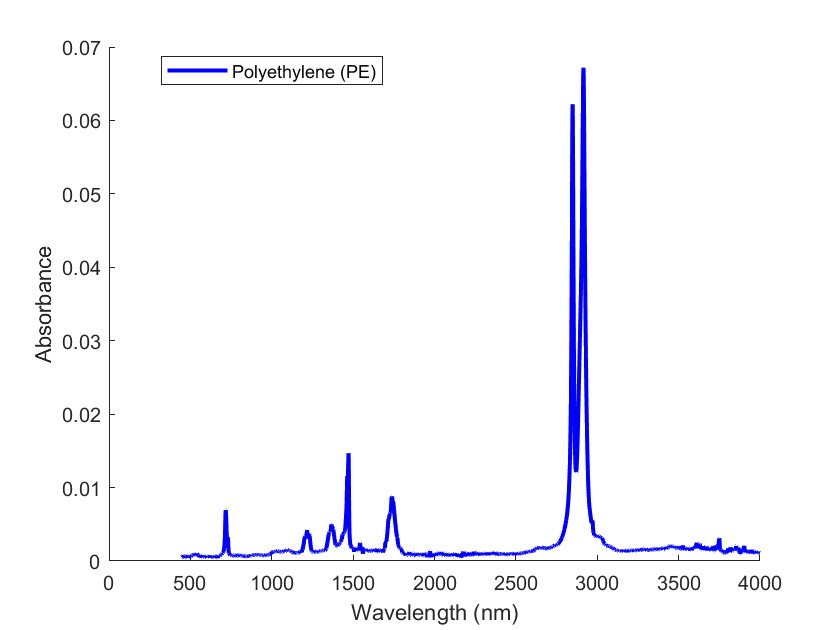


Supplementary Figure 10 Polyethylene peaks in Sludge 1

**
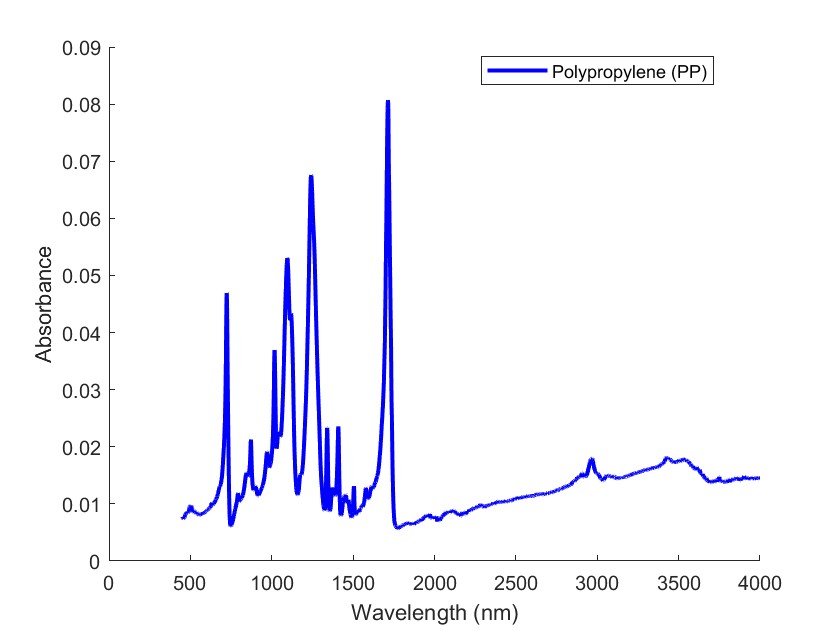
**

Supplementary Figure 11 Polypropylene peaks in Sludge 1


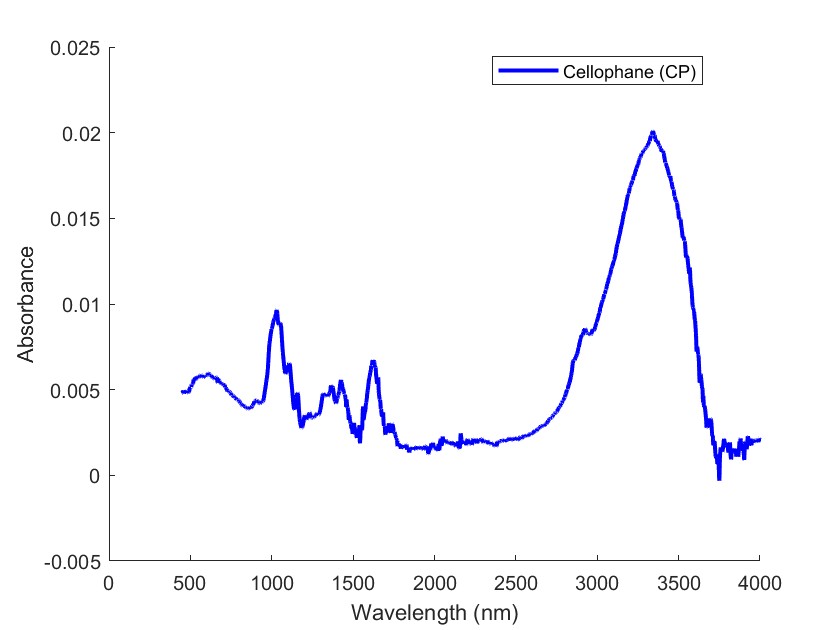


Supplementary Figure 12 Cellophane peak in Soil samples
